# Curcumin and Health

**DOI:** 10.3390/molecules21030264

**Published:** 2016-02-25

**Authors:** Mario Pulido-Moran, Jorge Moreno-Fernandez, Cesar Ramirez-Tortosa, MCarmen Ramirez-Tortosa

**Affiliations:** 1 Departamento de Bioquímica y Biología Molecular II, Facultad de Farmacia, Campus Universitario de Cartuja, Universidad de Granada, 18071 Granada, Spain; mpulido@ugr.es; 2 Instituto de Nutrición y Tecnología de los Alimentos José Mataix Verdú, Centro de Investigaciones Biomédicas, Avenida del Conocimiento s/n, Campus Tecnológico y Ciencias de la Salud, Universidad de Granada, Armilla (Granada) 18016, Spain; 3 Departamento de Fisiología, Facultad de Farmacia, Campus Universitario de Cartuja, Universidad de Granada, 18071 Granada, Spain; jorgemf@correo.ugr.es; 4 Servicio de Anatomía Patológica, Complejo Hospitalario de Jaen, 23007 Jaén, Spain; cesarl.ramirez.sspa@juntadeandalucia.es

**Keywords:** curcumin, bioavailability, antioxidant, ROS, anti-inflammatory, cancer, lung diseases, liver disorders, cardiovascular diseases, natural compound

## Abstract

Nowadays, there are some molecules that have shown over the years a high capacity to act against relevant pathologies such as cardiovascular disease, neurodegenerative disorders or cancer. This article provides a brief review about the origin, bioavailability and new research on curcumin and synthetized derivatives. It examines the beneficial effects on health, delving into aspects such as cancer, cardiovascular effects, metabolic syndrome, antioxidant capacity, anti-inflammatory properties, and neurological, liver and respiratory disorders. Thanks to all these activities, curcumin is positioned as an interesting nutraceutical. This is the reason why it has been subjected to several modifications in its structure and administration form that have permitted an increase in bioavailability and effectiveness against different diseases, decreasing the mortality and morbidity associated to these pathologies.

## 1. Introduction

Curcumin [1,7-bis(4-hydroxy-3-methoxyphenyl)-1,6-heptadiene-3,5-dione] also called diferuloylmethane, is the main natural polyphenol found in the rhizome of *Curcuma longa* (turmeric) and in others Curcuma spp. [1]. *Curcuma longa* has been traditionally used in Asian countries as a medical herb for several pathologies due to its antioxidant, anti-inflammatory [2], antimutagenic, antimicrobial [3,4], and anticancer properties [5,6].

In relation to the solubility properties, curcumin is soluble in alkali or in extremely acidic solvents [7]. It is a crystalline compound with a bright orange-yellow colour so it is used as food colorant [2]. It is a keto-enol tautomeric compound with a predominant keto-form in acid or neutral solutions and the enol-form is predominant in alkalis solutions with good properties as chelator of metal ions [8]. Different activities have been directly associated according to the keto or enol forms of curcumin [9].

**Figure 1:**
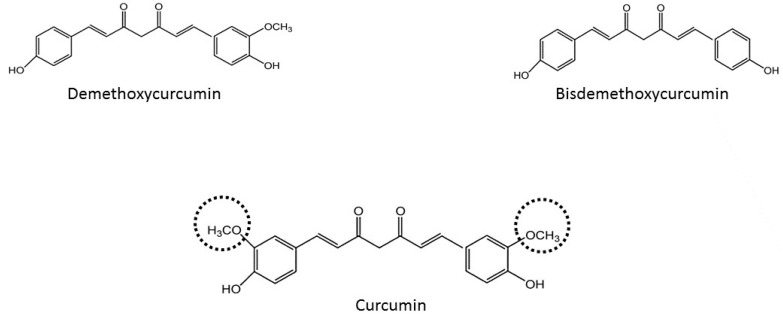
Chemical structures of curcuminoids (methoxy groups in curcumin are shown by discontinuous lines).

In the last 50 years it has been proven that most of the effects of *Curcuma longa* are mainly due to curcumin, with a potential effects against diabetes, allergies, arthritis, Alzheimer’s disease [10,11], and other chronic illnesses [9,12]. However, besides curcumin, there are others components in *Curcuma longa* called the curcuminoids group. They are demethoxycurcumin and bis-demethoxycurcumin. Curcumin is the most abundant of the curcuminoids group (77% of the total weight) [13] and this group constitutes around 5% of the total component of *Curcuma longa*. According to their structures (Figure 1), some researchers have concluded that the methoxy groups on the phenyl rings in curcumin are important to have health effects [14]. Moreover, it has been described that these curcuminoids have synergistic action for example to have nematocidal activity [15]. The aim of this review is to improve the knowledge of curcumin’s effects on health and try to elucidate its different mechanisms of action against different pathologies.

## 2. Bioavailability and Metabolism of Curcumin

In the last ten years, many studies with animals and *in vitro* models have been performed with the objective of stablishing the mechanisms of action of curcumin and its activities against several pathologies [16]. For this reason, there are many studies that aim to understand the bioavailability of curcumin. The first study performed to determine the biological availability was carried out by Wahlstrom and Blennow in 1978, where curcumin was administered to Sprague-Dawley rats at a 1 g/kg dose. In this study a low level of curcumin was observed in blood plasma of rats [17]. However, latter studies demonstrated that when curcumin was administered orally at a dose of 2000 mg/kg to rats, the maximum serum concentration was 1.35 ± 0.23 µg/mL although in humans it was undetectable [18].

Some studies with rats have shown that the oral bioavailability of curcumin was around 1% [19] so very high doses of curcumin are necessary (3600 to 12,000 milligrams) to achieve any beneficial effects. Sharman *et al.*, described no toxic effect of curcuma administered orally in patients with colorectal cancer [20]. This latter study showed that curcumin metabolites were detected in plasma when patients ingested at least 3600 milligrams of curcumin being mainly detected as curcumin glucuronide and curcumin sulphate. In urine samples, curcumin and its metabolites were detected primarily as curcumin, followed by glucuronide and finally as sulphate forms.

If curcumin is administered to rats in a dose of 2000 mg/kg jointly with l-piperoylpiperidine that induces glucuronyl transferase enzymes, the bioavailability of curcumin increases around 154% [18].

**Figure 2:**
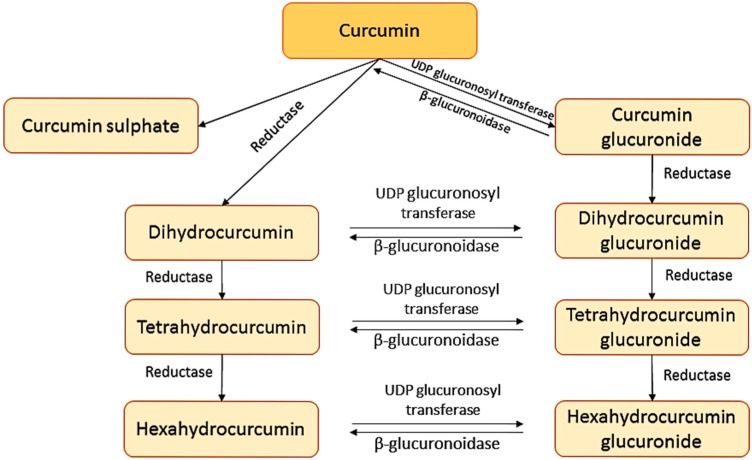
Metabolite derivatives of curcumin.

To determine the pharmacokinetic and pharmacodynamic properties of curcumin some researchers have measured the radioactivity after administration of [3H]-curcumin to rats, showing the great difficulty of curcumin in the intestinal absorption process [21,22,23]. In addition, these studies showed that curcumin suffers a biotransformation during the absorption from the bowel to glucuronides of tetrahydrocurcumin and hexahydrocurcumin derivatives. A further study was performed in liver to determine the nature of curcumin metabolites. These studies suggested that curcumin is firstly transformed to dihydrocurcumin and tetrahydrocurcumin by reductases and they are then transformed into monoglucuronide conjugates as dihydrocurcumin-glucuronide and tetrahydrocurcumin-glucuronide by β-glucuronidase [24]. The transformations that curcumin suffers are shown in Figure 2. The transformation in gut and liver can lead to generate curcumin glucuronides and curcumin sulphates or, alternately, reduced molecules like hexahydrocurcumin [25].

On the other hand, Ryu *et al.* [26] demonstrated that curcumin administered intravenously in mice as [18F]-curcumin was accumulated in the liver, spleen, lung and in brain. These authors concluded that curcumin has a specific affinity for some tissues.

Finally, the excretion of curcumin metabolites depends on the vehicle and the route of administration employed [27]. For oral administration, a 75% of curcumin metabolites were found only in faeces but not in urine [17]. However, when the curcumin was administrated intraperitoneally 73% of these metabolites were found in faeces and around 11% in urine [28]. In relation with the administration form, encapsulation in liposomes, polymeric nanoparticles, cyclodextrin encapsulation, lipid complexes or by synthesis of polymer-curcumin complex have been investigated. All of them have helped increase the activity and bioavailability [29] of this compound and to improve the beneficial effect of curcumin against some pathologies such as cancer [30,31] or liver diseases [32].

## 3. Role of Curcumin in Health

It is known that some diseases are produced by an unhealthy diet (*i.e*., cardiovascular diseases) and as Wood and Brooks suggest, “We are what we ate” [33]. In this sense, they are evidences that certain diets rich in antioxidants, as the Mediterranean, Indian or Nepalese diets, are excellent against some pathologies related with oxidative stress as cardiovascular diseases [34,35], cancer [36], metabolic disorders [37] or aging, improving the mortality and morbidity of them [38]. Particularly, many of the effects associated to Indian or Nepalese diets have been related with specific compounds such as curcumin and other curcuminoids [39].

### 3.1. Antioxidant Activity and ROS Scavenger Properties

Curcumin is considered as antioxidant due to the β-diketone group in its structure [14,40,41]. Joe and Lokesh determined in 1994 that the most important mechanisms by which curcumin is able to promote the majority of its activities are by inhibition of superoxide radicals, hydrogen peroxide and nitric oxide radical [42]. Other studies proposed that curcumin also enhances the activity of many antioxidant enzymes such as catalase, superoxide dismutase (SOD), glutathione peroxidase (GPx) [43] and heme oxygenase-1 (OH-1) [44]. These activities reduce the lipid peroxidation decreasing the hepatic damage [45,46]. In addition, curcumin is also able to increase the activity of xenobiotic detoxifying enzymes both in liver and kidneys, protecting against carcinogenesis processes [47].

Other studies have described that a dose of 200 mg/kg of curcumin increased the SOD and catalase activities and the hepatic total antioxidant capacity in liver from rats with hepatic damage and high level of oxidative stress provoked by thallium acetate [48].

In human hepatocyte L02 cell line curcumin was able to avoid the ROS formation by increasing SOD activity and reducing glutathione levels after treatment with the antimicrobial feed additive quinocetone as a generator of free radicals [49].

**Figure 3:**
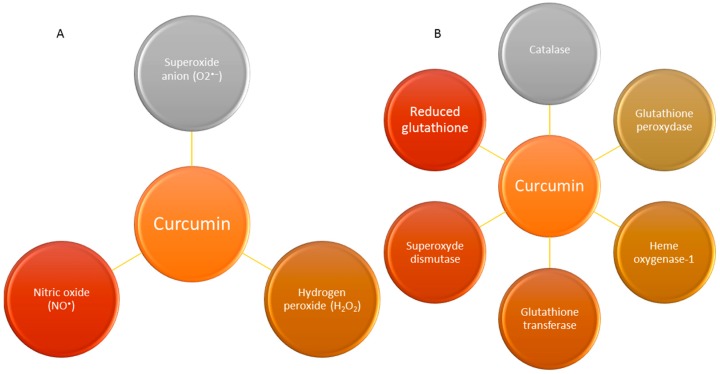
This figure shows a brief summary of the antioxidant properties and ROS scavenger effects of curcumin. (**A**) Effect of curcumin as ROS and RNS scavengers; (**B**) Enhanced activities of curcumin on different antioxidant systems.

In addition, curcumin can upregulate other enzymes like glutathione transferase and their mRNAs as well as increase both the level of reduced glutathione and the acid-soluble sulphydryl groups [50] and scavenge free radicals [51]. All these effects together have granted it a health- and radioprotective role [42,45,52,53,54]. Moreover, curcumin can increase the levels of reduced gluthathione (GSH) and it can moderate the malondialdehyde levels in a lung carcinogenesis model induced by benzo(a)pyrene (a major carcinogenic pollutant) in mice [55]. The main antioxidant activities and ROS scavenger properties of curcumin in the biological system are described in Figure 3.

Some studies have determined that curcumin is able to prevent the brain injury thanks to the suppression of oxidative stress via Akt/Nrf2 (Nuclear factor-E2-related factor 2) pathway, acting then as neuroprotector [56]. Li *et al.* [57] proved the neuroprotector effect of curcumin by decreasing the reactive oxygen species (ROS)-associated endoplasmic reticulum (ER) stress (related to neuronal damage) through the regulation of AMPK activity. Similar results were obtained by Fu, Yang *et al.* [58] who tried to determine if curcumin could protect PC12 cells from H_2_O_2_-induced neurotoxicity, These authors described that curcumin dysregulated the mitogen-activated protein kinase (MAPK) and AKT pathways, decreasing apoptotic cells through inhibition of ROS accumulation [58].

Curcumin expresses a dichotomy between its antioxidant and pro-oxidant effects, generally depending on the dose used and the presence in the medium of metal ions [50,59,60]. Curcumin can selectively provoke the induction of pro-oxidant effects only in malignant cells for example in cervical cancer cells [61], cell lung cancer NCI-H446 [62] or in murine myelomonocytic leukemia WEHI-3 cells [63]. These studies described that curcumin promotes a powerful increase in the intracellular generation of ROS or ER-stress by provoking a mitochondrial dysfunction.

### 3.2. Curcumin and Inflammation

The inflammatory cascade plays an important role in the development of chronic illnesses such as autoimmune, cardiovascular, endocrine, neurodegenerative and neoplastic diseases [64,65,66]. Curcumin is able to decrease inflammation by interacting with many inflammatory processes [67,68,69,70].

Oxidative stress can lead to chronic inflammation, causing chronic diseases [71]. ROS production modulates the expression of thebnuclear factor-κβ (NF-κβ) and tumour necrosis factor alpha (TNF-α) pathways which play a central role in the inflammatory response [72]. Curcumin could downregulate oxidative stress and the subsequent inflammation through the Nrf2 pathway. This polyphenol can decrease TNFα production and the cell signalling mediated by TNFα in various types of cells. *In vitro* and *in vivo* studies postulate that curcumin is a TNFα blocker due to its direct union to TNFα [73,74]. In this sense, curcumin modulates TNF-α expression by inhibition of p300/CREB-specific acetyltransferase which leads to repression of acetylation of histone/nonhistone proteins and therefore repression of transcription [74]. Regarding NF-κβ the primary transcription factor involved in the start of the inflammatory processes, can be blocked by curcumin [75]. This molecule inhibits the IkBkinase provoking a NF-κβ inhibition.

Curcumin also inhibits inflammatory cytokines, such as interleukins (ILs), chemokines, as well as inflammatory enzymes, such as cycloxygenase-2 (COX-2), inducible nitric oxide synthase (iNOS) and others molecules as cyclinD1 [76]. This natural anti-inflammatory agent is able to inhibit MAPK and NF-κB pathways in TNF-α-treated HaCaT cells and, therefore, IL-1β and IL-6 expression [77]. In others studies in BV2 microglia cell stimulated with lipopolysaccharide (LPS), curcumin also inhibited IL6 and TNF-α [78]. Due to all its anti-inflammatory properties, curcumin is able to improve different chronic diseases as follows.

#### 3.2.1. Inflammation of Respiratory System

Allergy is a pro-inflammatory disease mediated by cytokines [79]. In asthma development, others inflammatory molecules such as eotaxin, monocyte chemoattractant protein-1 (MCP-1) and MCP-3 play also an important role. In this sense, in 2015 Panahi *et al.*, studied the anti-inflammatory properties of curcuminoides in sulphur mustard-intoxicated subjects. These authors found a great effect of curcuminoides *vs.* placebo in modulating all assessed inflammatory mediators included MCP-1 [80].

There are many studies which confirm the important role of curcumin in respiratory diseases caused by inflammation [81]. In acute allergic asthma in BALB/c mice, the anti-inflammatory effect of curcumin, the down-regulated levels of Notch1/2 receptors and globin transcription factor 3 (GATA3) was reported [82]. Studies of plants extracts with anti-asthmatic components showed that curcumin can act as a scavenger of nitric oxide and could prevent bronchial inflammation in asthmatic patients [83]. Moreover, curcumin can also decrease allergic inflammation in asthma models by regulating Treg/Th17 balance with an important increase of Treg cells [84].

#### 3.2.2. Inflammation of Joints

ROS play a crucial role in joint destruction in rheumatoid arthritis [85,86]. These free radicals can activate many transcription factors such as NF-κβ and activator protein 1 (AP-1), which regulate growth factors, chemokines, inflammatory cytokines and anti-inflammatory molecules [87]. Curcumin as an anti-inflammatory and antioxidant compound possesses anti-rheumatic and anti-arthritic properties [88,89]. In patients with rheumatoid arthritis, curcumin is able to up regulate pro-apoptotic Bax, and down regulate anti-apoptotic B-cell lymphoma 2 (Bcl-2) and X-linked inhibitor of apoptosis protein (XIAP), inducing apoptosis and, therefore, inhibit the growth of synovial fibroblasts [90]. Curcumin also prevents the antiinflammatory response in synovial fibroblasts by inhibition of prostaglandin E2 (PGE2) synthesis due to COX-2 suppression [91].

#### 3.2.3. Digestive System Inflammation 

Inflammatory bowel disease is characterized by oxidative stress, nitrosative stress, leukocyte infiltration and production of pro-inflammatory cytokines. NF-κβ also takes part in this disease producing cytokines and chemokines for inflammation [92]. Curcumin is able to suppress STAT3 pathways, reducing the expression of TNFα, and IL-1β. Moreover, this molecule can improve dextran sulphate sodium (DSS)-induced colitis because it decreases myeloperoxidase activity, colon injury, oxidative stress, inflammatory reaction, and apoptotic cell death by blocking c-Jun N-terminal protein kinase (JNK), p38 MAPK pathways [93,94]. According to this, other studies showed that curcumin blocked p38 MAPK and Akt pathways, decreased NF-κβ level and inhibited the expression of vascular cell adhesion molecule 1 (VCAM-1) in human intestinal microvascular endothelial cells (HIMECs) and in 2,4,6-trinitrobenzenesulphonic acid-induced colitis in mice [95,96,97].

In others digestive diseases such as chronic pancreatitis many factors can participate (alcohol tobacco, genetic, environmental, hypertriglyceridemia, hypercalcemia, autoimmune) [98]. Curcumin reduces inflammation by decreasing NF-κβ, AP-1, iNOS, TNF-α, and IL-6 molecules in rats with pancreatitis [99]. In pancreatitis induced by ethanol and cerulein, curcumin improves disease’s severity, acting on serum amylase, pancreatic trypsin, and neutrophil infiltration [100]. Dhillon *et al.*, [101] described that curcumin has biological activity in patients with pancreatic cancer. Curcumin down-regulated expression of NF-κβ and COX-2.

Some studies evaluated the effect of curcumin in gastric mucosa inflammation provoked by *Helicobacter pylori*. In these studies, curcumin decreased the levels of inflammatory cytokines, chemokines, the expression of toll-like receptors (TLRs) and myeloid differentiation primary Response 88 (MyD88) in mice. These results indicate that curcumin exerts an anti-inflammatory effect in *H. pylori*-infected mucosa [102].

### 3.3. Curcumin in Neurological Disease

The World Health Organization (WHO) has defined neurological disorders as different pathologies of the central and peripheral nervous system, including the brain, spinal cord, cranial nerves, peripheral nerves, nerve roots, autonomic nervous system, neuromuscular junction, and muscles. These disorders produce epilepsy, Alzheimer disease (AD), Parkinson’s disease (PD), migraine, brain tumors and traumatic disorders of the nervous system.

Curcumin can interact and modulate many molecular targets such as transcription factors, inflammatory cytokines, kinases, growth factors and antioxidant system. All these actions of curcumin will be responsible, at least in part for its neuroprotective. Many of these beneficial effects mentioned before have been demonstrated by several paths in different types of cells from the nervous system, such as neurons [103], astrocytes [104] and microglia [105]. Some researchers employing primary cell cultures from different regions of the nervous system have also observed the capacity of curcumin to act as neuroprotector due to its antioxidant, anti-inflammatory and anti-protein aggregating properties [106] in cortical [105], mesencephalic [107], hippocampal [108] and spinal cord cells [109].

Furthermore, the capacity of curcumin to decrease neuro-inflammation, which takes place in the progression of neurodegenerative diseases, by reducing the expression of IL-1α, IL-6 and TNF-α in LPS-stimulated BV2 microglia in a dose dependent manner has been well established [77]. AD shows signs of defective phagocytosis that results in an ineffective clearance of Aβ plaques. Curcumin can stimulate microglial phagocytosis and clearance of Aβ plaques *in vitro* and increase the induction of heat-shock proteins in response to the addition of soluble Aβ aggregates to neuronal cell cultures [110]. It also can attenuate the β-amyloid accumulation and inhibit the formation of Aβ fibrils in these cells [111]*.* Moreover*,* in dopaminergic neurons, neurotoxicity triggered by 6-hydroxydopamine (6-OHDA) curcumin attenuated the toxicity in SH-SY5Y and MES23.5 cells through the inhibition of ROS, mitochondrial protection and anti-apoptotic mechanisms [112,113].

Several studies in rodents have proved the neuroprotection of curcumin in neurodegenerative disorders, especially AD and PD. Curcumin can also attenuate oxidative injury, microgliosis, synaptophysin loss, spatial memory deficits, postsynaptic loss, and Aβ deposits produced by intra-cerebral ventricular infusion of Aβ amyloid in rats [114]. Moreover, a study performed in Tg2576 mice (a transgenic mouse model for AD) fed curcumin described a decrease of amyloid plaque formation ROS and reactive nitrogen species (RNS) formation [115]. Finally, in mice overexpressing Aβ (PS1dE9) where curcumin was intravenously administered, a plaque disruption and an attenuation of distorted neuritis were found [116].

In regards to PD, other studies have tested the neuroprotective potential of curcumin against 1-methyl-4-phenyl-1,2,3,6-tetrahydropyridine (MPTP) and 6-OHDA-induced dopaminergic degeneration. Oral and intravenous administration of curcumin were able to modulate dopaminergic damage in 6-OHDA-treated rodents, suppressing apoptosis, inducing microglial activation and improving the locomotion [117]. Other studies performed with CAG140 mice described a powerful capacity of curcumin to decrease Huntington protein aggregation, improving rearing deficits, but impairing climbing behavior [118]. A study in β-amyloid-infused rats showed that curcumin improved spatial memory [119]. Mechanisms through which curcumin can improve memory in AlCl_3_-challenged mice is decreasing oxidative stress and histopathological damage in brain [120,121].

### 3.4. Role of Curcumin in Lung Injuries

Respiratory diseases are a complex group of disorders in the respiratory system, from the upper tract into the pleural cavity, including both muscles and the nervous systems that modulates it. The most studied pulmonary pathologies are fibrosis, bronchitis, allergy and asthma in relation with inflammatory process and redox status alterations. The effects of curcumin in these process are decreasing inflammatory cells accumulations [122], cytokines over-production [123] and increasing ROS scavengers [52].

In 1996, the first evidence that curcumin has effects against pulmonary injuries was described by Thresiamma *et al.* [124] using a radioactive curcumin administered orally in mice. Further studies demonstrated the effect of curcumin in many pulmonary injuries [125,126,127].

Pulmonary fibrosis is characterized by an accumulation of inflammatory cells in the airways [125]. These cells can generate a high increase in the cytokines, ROS and growth factors which promote in turns the basis of the fibrotic scar. In this sense, several studies performed in animal models have demonstrated that curcumin acts against pulmonary fibrosis mainly by decreasing the inflammatory mediators [126,127]. Venkatesan and Chandrakasa [128] showed the beneficial effects of curcumin against cyclophosphamide-induced pulmonary fibrosis in rats. These effects were a reduction in leukocytes and inflammatory mediators and an increment in lung antioxidant defences. Similar results were obtained by Venkatesan [129] who also described that rats injected with paraquat, to provoked pulmonary fibrosis and treated with curcumin had a lower inflammatory response, toxicity and mortality associated to this herbicide.

Asthma is a chronic inflammatory disease identified by a reversible airflow obstruction and bronchospasm with variable intensity. Asthma is also characterized by goblet cell hyperplasia, airway eosinophilia, mucus hypersecretion and hyperresponsiveness to endogenous and exogenous allergens [130]. Several transcription factors are involved in the inflammatory process of asthma, including the NF-κβ, AP-1, cyclic AMP response element binding protein, peroxisome proliferator-activated receptor (PPAR) and the bZIP transcription factor, Nrf2 [131,132]. Eosinophils play a critical role in asthma by producing inflammatory mediators and free radicals [133]. In this sense, there are critical interleukins involved in the activation process of eosinophils such as IL-5, as well as IL-4 but in a lesser degree [134]. Their control can be considered as a novel strategy against asthma [135].

Curcumin has been widely used in ancient Indian medicine for allergy and asthma treatment [81] and currently, several studies have found that curcumin has a powerful activity both *in vitro* and *in vivo* against asthma. These effects are produced by an inhibition of IL-5, IL-4, IL-2, immunoglobulin (Ig) E2 production [136,137] and by decreasing the histamine effects in peritoneal mast cells from rats [138,139].

In addition, Oh *et al.* [132] demonstrated the ability of curcumin to inhibit NF-κβ production in a dose-dependent form by the inactivation of Iκ-Bα and AKT in pathogen-free BALB/c mice and in murine macrophage-like Raw264.7 cell line [67,140].

Other studies have described that curcumin can improve the elimination of nitric oxide and decrease the nitric oxide synthase activity. This might be a mechanism of curcumin which could prevent the bronchial inflammation in asthmatic patients [83,141]. In addition, curcumin can protect against asthmatic mucus secretion and airway hyperresponsiveness through an increment of Nrf2 and HO-1 levels in lung [94]. In summary, all these beneficial effects of curcumin against asthmatic pathologies and others lung injures immune-, ROS- and inflammation-associated [142,143,144].

### 3.5. Relationship between Metabolic Syndrome and Curcumin

According to the American Heart, Lung and Blood Institute (NHLBI) metabolic syndrome is defined as a “group of risk factors that raises your risk for heart disease and other health problems, such as diabetes and stroke”. All risk factors are closely linked to obesity, to insulin resistance, lifestyle and diet. For this reason the metabolic syndrome is also called “The Deadly Quartet” of hyperglycaemia, hypertriglyceridemia, hypertension, and obesity [145] and the concurrence of all these together for a long time, finally leads to cardiovascular diseases and diabetes type II [146].

Currently, inflammation is recognized as an overwhelming burden on healthcare status [147]. It is known that an increase of pro-inflammatory molecules (TNF-α, IL-6, MCP-1, and IL-1) directly secreted from adipocytes is related to insulin resistance and chronic inflammation [148,149].

In the study performed by Rains *et al.* [150], the authors observed that curcumin has immunomodulatory effects in obesity and insulin resistance because it decreases cytokines, TNF-α, MCP-1, glucose and glycosylated haemoglobin in diabetic rats [151,152].

Another study employing C57BL/Ks-db/db diabetic mice demonstrated that curcumin decreased blood glucose and glycosylated haemoglobin levels and increased the plasma insulin levels and hepatic glucokinase activity. In addition, curcumin decreased the glucose-6-phosphatase and phosphoenolpyruvate carboxykinase activities reducing glucose levels in blood [153] and improving the glucose tolerance.

On the other hand, curcumin also decreased plasma free fatty acid, cholesterol, and triglyceride levels and increased hepatic glycogen and skeletal muscle lipoprotein lipase (LPL) [153,154]. Kaur *et al.* [155] in a study with an extract of curcumin, piperine and quercetin (both of them to enhance the bioavailability of oral curcumin) showed that diabetic rats fed a high fat diet showed decreased plasmatic levels of glucose, triglycerides, total cholesterol and low density lipoprotein (LDL) with a concomitant increase high density lipoprotein (HDL).

Moreover, the decrease in plasma free fatty acid by curcumin in diabetes also promotes lower lipotoxicity, considered as a trigger of insulin resistance through activation of NF-κβ [156]. In relation to obesity, curcumin reduces body weight gain and angiogenesis in adipose tissue, decreases pre-adipocytes differentiation and lipid accumulation in mature adipocytes in mice [157] favouring the non-appearance of one of the risk factors of metabolic syndrome triggers [158,159].

All these results corroborate the powerful activities of curcumin appeasing hyperlipidaemia, insulin resistance and glucose tolerance associated with excess dietary fat intake, obesity, and type 2 diabetes [155,160].

Finally, several clinical trials have been performed in humans with metabolic syndrome employing both curcumin and curcuminoids. In these studies curcumin reduced lipid profile and modified cholesterol-related parameters [161,162]. Furthermore an study suggests the relevance of curcumin improving the anthropometric measurements and body composition when it is associated to diet and lifestyle intervention [163].

### 3.6. Curcumin as Hepatoprotective

The liver is intensely involved in the metabolism and synthesis of all macronutrients and it presents highly relevant endocrine and exocrine functions. The liver is also responsible for the detoxification of the blood. The liver has a very complex architecture and present different types of cells such as hepatocyte, cholangiocyte/bile duct cell, endothelial cell, liver sinusoidal endothelial cell, pit cell, Kupffer cell and hepatic stellate cell [5,164]. All activities performed by the liver are highly relevant for life and for this reason, when liver diseases appear, these functions are substantially compromised, with a resulting increase in morbidity and mortality [164].

The beneficial effects of curcumin in liver diseases can be due to its anti-inflammatory, antioxidant effects and antifibrogenic properties [165]. In addition, curcumin can decrease levels of thiobarbituric acid reactive substances (TBARS) and increase GSH and SOD levels in the liver homogenates from LPS-challenged rats supplemented with curcumin [166]. Curcumin also reduces the iron-induced hepatic damage by lowering lipid peroxidation [45,46], increase the activity of xenobiotic detoxifying enzymes [47] and the hepatic total antioxidant capacity [48]. Finally, this molecule can up regulate the cytoprotective enzyme HO-1 [167] inhibiting the ROS formation in liver [49].

In this sense, some studies carried out by our research group [168] have demonstrated that curcumin decreased the non-alcoholic steatohepatitis (NASH) and aminotransferase activity, and increased the mitochondrial antioxidants in rabbits with high-fat-induced NASH. Moreover, curcumin also reduced mitochondrial ROS, improved mitochondrial function, and lowered levels of TNF-α. All these results were corroborated by Wang *et al.* [169] in a rat model.

According to the hepatoprotective effects of curcumin, there are studies which have described a down-regulation of NF-κβ transcription factor [170], an improvement of hepatic fibrosis in alcoholic liver injuries [171], an increase of the survival rates in animals [172] and a reduction of damage in experimental steatohepatitis [173].

### 3.7. Has Curcumin Got an Antitumor Effect?

Regarding the antitumor properties of curcumin, they are a lot of studies carried out in animals, human leukaemia cell types [12], and several clinical trials in cancer patients. Only few of them have described the anticancer potential of curcumin [20,174] and many of them have investigated the capacity of curcumin as an adjuvant in anti-tumour therapy or reducing adverse effects associated with the treatment [174,175,176,177,178]. In this sense, Sharma *et al.* [179] showed that curcumin prevent colon cancer in rodent model by inhibition of lipid peroxidation and cyclooxygenase-2 (COX-2) expression and by increase of glutathione S-transferase (GST) enzymes [180].

**Figure 4:**
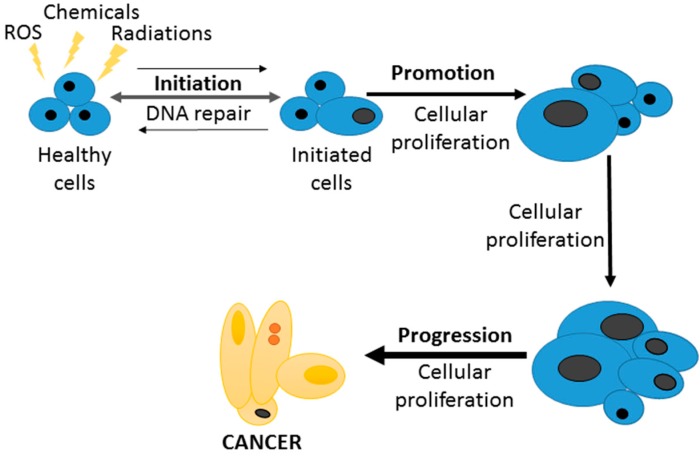
The three steps in the carcinogenesis process: initiation, promotion and progression.

On the other hand, ROS and inflammation are linked to carcinogenesis processes [71,181], mainly acting as initiators in the well-known triphasic theory of cancer: initiation, promotion and progression (Figure 4). For this reason, some anticancer properties of curcumin are due to the antioxidant and ROS scavenger activities already described before. Moreover, the effects of curcumin in different genes and proteins such as Bcl-2, VCAM-1, Cyclin D1, Bax, NF-κβ, VGEF or COX-2, curcumin prevents the promotion and progression stages [182]. Hosseini and Ghorbani [183] have described the anticancer effects of curcumin by multiple actions on mutagenesis, cell cycle regulation, apoptosis and oncogene expression. However, curcumin also can modulate different pathways related to angiogenesis, invasion, tumour growth or metastasis [184] and it can promote apoptosis through interaction with p53 [185], caspase expression [186], and inducing cell cycle arrest [187].

Several epidemiological studies have determined that inflammation is one of the most important risk factors associated to carcinogenesis. During this process, the activation and interaction between NF-κβ and STAT3 has been identified as a key mechanism in the communication between cancer cells and inflammatory cells [188]. As has already been described previously, curcumin is able to down-regulate different pathways related to both molecules, then decreasing the generation of different inflammatory mediators such as COX-2, lipoxygenase 2 (LOX-2), iNOS, and cytokines associated to them and hence preventing the carcinogenesis process in several types of cancer [189,190,191].

In addition, curcumin inhibits JAK-STAT pathway [192] and inhibits telomerase activity [193], finally driving to the arrest of cell cycle and apoptosis in tumour cells.

Regarding to breast cancer, curcumin has demonstrated similar results by inhibition of COX-1, COX-2, Bax expression, vascular endothelial growth factor (VEGF) and the telomerase activity [71,194,195]. Curcumin also downregulates the inflammatory cytokines CXCL1 and CXCL2 by the inhibition of NF-κβ [196].

In human lung cancer cells, curcumin has similar mechanisms decreasing the migration and invasion of cells through inhibition of MMP-2 and MMP-9 and suppression of VEGF expression [197]. In this sense, Das and Vinayak [198] described how curcumin regulates genes as hypoxia-inducible factor 1-alpha (HIF-1a) and MYC, LDH activity and decreases the angiogenesis process by a MMP-2, MMP-9, PKC-a and VEGF reduction. All these results are in accord with studies performed in a lung cancer mice model which also showed that curcumin can inhibit the neutrophil chemoattractant keratinocyte-derived chemokine expression (CXC-KC) stopping the tumour progression [199].

Finally, several cytokines have been focused as target for prevention and treatment of several types of tumours [200,201,202]. In this sense, curcumin can inhibit some of these cytokines such as IL-1β, IL-6, TNFα or IL-23 so this molecule has an important role in cancer that overexpress them such as oral cavity, forestomach, duodenum, and colon [203].

### 3.8. How Does Curcumin Prevent Cardiovascular Pathologies?

According to the definition proposed by the World Health Organization, cardiovascular diseases (CVDs) are a complex group of disorders that affect the heart and blood vessels. These disorders are associated with risk factors such as an unhealthy diet, physical inactivity, tobacco use and use of alcohol. There are many signs and symptoms of these disorders as raised blood pressure, raised blood glucose, raised blood lipids, and excess weight and obesity. One of the most important causes of stroke and heart attacks is atherosclerosis. This is a complex and multistep process with many factors involved in the formation and evolution of the atherosclerotic plaque. Lipoprotein oxidation and oxidative processes play a critical role in the pathogenesis atherosclerosis. Some studies have identified the oxidized LDL (ox-LDL) as a powerful atherogenic agent [204,205].

Several mechanisms have been proposed by which ox-LDL can develop the atherogenic process such as the enhanced uptake by macrophages which leads to foam-cell formation, cytotoxic actions and alteration of platelet aggregation mechanism [206,207]. In this sense, some studies performed by our research group, have demonstrated the effectiveness of a curcumin extract to downregulate the LDL and ox-LDL concentration and also increase the HDL level by decreasing the oxidative stress and attenuating aortic fatty streak development in a rabbits fed a high cholesterol diet [208,209]. In addition, curcumin also exhibits an inhibitory effect on the platelet aggregation and eicosanoid metabolism, showing its cardio protective character [210]. In a coronary artery disease study performed in humans, curcumin was able to decrease, very low density lipoprotein (VLDL), LDL cholesterol and serum triglyceride with no effect on inflammatory biomarkers [211].

The strong association between cardiovascular diseases risk and inflammation has been well established [207,212,213]. As has been described before, curcumin decreases the levels of inflammatory molecules such as TNF-α, p38 MAPK, JAK2/STAT3 so it improves cardiovascular risk inflammation-associated [214,215]. Moreover, curcumin suppress the activation of TLR4, inhibits phosphorylation of ERK1/2 and nuclear translocation of NF-κβ and reduces nicotinamide adenine dinucleotide phosphate (NADPH)-mediated intracellular ROS production [216].

In relation to cardioprotective effects associated to oxidative stress, curcumin attenuates oxidative stress by up-regulating eNOS expression, decreasing p47phox NADPH oxidase and superoxide generation the vascular tissues. All these effects promote a descent of blood pressure and hind limb vascular resistance and increased hind limb blood flow [217]. In addition, curcumin increases Nrf2 expression and inhibits NF-κβ activation in an obesity-induced heart injury both in *in vivo* and *in vitro* models [218]. In 5/6 nephrectomised rats curcumin also promoted the Nrf2 activation, and prevented of hemodynamic changes and glomerular hypertension [219,220]. In the study carried out by Abo-Salem *et al.* [221], curcumin increased cardiac antioxidant enzymes such as catalase, SOD, glutathione-S-transferase and glutathione reduced in streptozotocin-induced heart injury in rats.

Finally, curcumin can also act as cardioprotector in an ischemia reperfusion injury model. This effect is produced because curcumin can activate SIRT1 signalling which reduces mitochondrial damage. SIRT1 reduces some molecules as SOD, succinate dehydrogenase, cytochrome c oxidase, methane dicarboxylic aldehyde and H_2_O_2_ levels in mitochondria [222]. In summary, the natural cardioprotective activity of curcumin is in part due to a reduction of the oxidative stress.

## 4. Conclusions

Through this review, the beneficial effects of curcumin against different diseases highly relevant in modern societies has been proved. In summary it can be concluded that curcumin has protective health effects mainly through anti-inflammatory and antioxidant mechanisms. Thus, curcumin has an important role against neurological, cancer, cardiovascular and lung diseases, and also against metabolic syndrome and liver disorders. Likewise, several specific mechanisms of action have been postulated for curcumin, to understand its beneficial effects and its role as a nutraceutical compound.
